# Deficiency for the Chemokine Monocyte Chemoattractant Protein-1 Aggravates Tubular Damage after Renal Ischemia/Reperfusion Injury

**DOI:** 10.1371/journal.pone.0123203

**Published:** 2015-04-13

**Authors:** Ingrid Stroo, Nike Claessen, Gwendoline J. D. Teske, Loes M. Butter, Sandrine Florquin, Jaklien C. Leemans

**Affiliations:** Department of Pathology, Academic Medical Center, University of Amsterdam, Amsterdam, The Netherlands; University of Kentucky, UNITED STATES

## Abstract

Temporal expression of chemokines is a crucial factor in the regulation of renal ischemia/reperfusion (I/R) injury and repair. Beside their role in the migration and activation of inflammatory cells to sites of injury, chemokines are also involved in other processes such as angiogenesis, development and migration of stem cells. In the present study we investigated the role of the chemokine MCP-1 (monocyte chemoattractant protein-1 or CCL2), the main chemoattractant for monocytes, during renal I/R injury. MCP-1 expression peaks several days after inducing renal I/R injury coinciding with macrophage accumulation. However, MCP-1 deficient mice had a significant decreased survival and increased renal damage within the first two days, i.e. the acute inflammatory response, after renal I/R injury with no evidence of altered macrophage accumulation. Kidneys and primary tubular epithelial cells from MCP-1 deficient mice showed increased apoptosis after ischemia. Taken together, MCP-1 protects the kidney during the acute inflammatory response following renal I/R injury.

## Introduction

Chemokines are important players in the regulation of inflammatory and subsequently reparative processes taking place after renal ischemia/reperfusion (I/R) injury. Among them, monocyte chemoattractant protein-1 (MCP-1) also known as CCL2, is believed to play an important role. MCP-1 is produced by tubular epithelial cells (TEC) in response to various stimuli (reviewed in[1]). As early as 1991, Safirstein *et al*. reported an increased and prolonged expression of MCP-1 after renal ischemia[2]. Importantly, MCP-1 expression was reported to correlate with monocyte infiltration in the post-ischemic kidney[3]. Furthermore, in vitro studies using various human TEC cell lines have shown that the majority of monocyte chemoattractant activity produced by these cells is accounted for MCP-1[4;5].

*In* vivo MCP-1 is the main chemoattractant for monocytes; MCP-1 deficiency results in a decreased and delayed macrophage accumulation in various inflammatory models[6–9]. Macrophages can be roughly divided into either classical (M1) or alternate (M2) activated, possessing a pro-inflammatory and an anti-inflammatory/tissue remodeling phenotypic function respectively (reviewed in[10]). There is a tight balance between the detrimental and beneficial effects of macrophages; reducing macrophage accumulation does not necessarily lead to better outcome after an inflammatory event. Indeed, in a model of skeletal muscle I/R[8] and in myocardial infarct[6] reduced numbers of macrophages are accompanied by an accumulation of (apoptotic) neutrophils and damaged cells in MCP-1^-/-^ mice suggesting defective phagocytosis of dead cells and impaired tissue remodeling.

Moreover, the role of MCP-1 extends beyond its monocyte chemoattractant properties. MCP-1 activates the respiratory burst of monocytes and induces the expression of the pro-inflammatory cytokines IL-6 and IL-1β[11]. In addition, MCP-1 may exert direct effects on TEC; *in vitro* MCP-1 triggers TEC leading to activation of NFκB, a transcription factor commonly involved in inflammatory responses, without interaction with its chemokine receptor CCR2[12]. Based on these properties MCP-1 can be regarded as pro-inflammatory. On the other hand, MCP-1 inhibits apoptosis of T cells[13], cardiomyoctes[14;15], neurons and astrocytes[16], alveolar epithelial cells[17], and prostate cancer cells[18].

Studies exploring the role of MCP-1 in renal I/R injury have focused on the interaction between MCP-1 and its chemokine receptor CCR2. Decreased renal damage and reduced influx of macrophages and neutrophils was observed after blocking CCR2 in renal I/R injury[19;20]. However, in addition to MCP-1, the monocyte chemoattractant proteins MCP-2, -3, and -5 are also chemokine ligands for CCR2[21]. Therefore, blocking CCR2 does not discriminate between the different monocyte chemoattractants. In addition, since recent data indicates that MCP-1 has versatile functions which might be independent of interaction with CCR2, we wanted to investigate the role of this chemokine in ischemic injury in the kidney. For this, we induced renal I/R injury in MCP-1^+/+^ and MCP-1^-/-^ mice and analyzed the subsequent pathophysiology. We demonstrate that MCP-1 deficiency increased lethality and renal damage following renal I/R injury. Moreover, both *in vivo* and *in vitro*, MCP-1 deficiency enhanced apoptosis of tubular epithelial cells.

## Materials and Methods

### Mice

Eight till twelve-week old male C57Bl/6J mice were purchased from Charles River (Maastricht, the Netherlands). MCP-1^-/-^ mice, backcrossed to a C57Bl/6J genetic background for 10 generations, were obtained from Jackson Laboratory (Bar Harbor, Maine) and bred in the animal facility of the Academic Medical Center in Amsterdam, The Netherlands. Mice were housed under specific pathogen-free conditions receiving food and water *ad libitum*. Age- and sex-matched mice were used in all experiments. All experimental procedures were approved by the local Animal Care and Use Committee of the University of Amsterdam, the Netherlands (Permit number DPA100947 and DPA102258). All surgery was performed under general anesthesia and mice received buprenorphine for analgesic purpose. All efforts were made to minimize suffering.

### Renal I/R Injury Model

Bilateral renal I/R injury was induced by clamping both renal arteries for 30 minutes under general anesthesia (0.07 mg/10 g mouse of fentanyl citrate fluanisone midazolam mixture, containing 1.25 mg/ml midazolam [Roche, Mijdrecht, the Netherlands], 0.08 mg/ml fentanyl-citrate, and 2.5 mg/ml fluanisone [Janssen Pharmaceuticals, Beerse, Belgium]) as described before[22]. After removal of the clamp, the kidneys were inspected for restoration of blood flow. For analgesic purposes, mice received a subcutaneous injection of 50 μg/kg buprenorphin (Temgesic; Schering-Plough, Brussels, Belgium) after closing the abdomen. Sham-operated mice underwent the same procedure except clamping of the renal arteries. Mice were sacrificed 1 day after surgery. At the time of sacrifice, blood was collected by heart puncture in heparin-containing tubes and stored at -80°C, and kidneys were harvested for further analysis. In the survival study MCP-1^+/+^ and MCP-1^-/-^ mice (n = 22) were subjected to bilateral renal I/R injury as described before and monitored three times a day up to 14 days after surgery. Mice that reached the humane endpoint, separated themselves from the group and were unable to right themselves within 5 seconds, were euthanized (2–3% isoflurane in 100% O_2_ anesthesia followed by cervical dislocation) immediately and recorded as dead.

### Plasma biochemical analysis

Using standard autoanalyzer methods plasma levels of ureum, creatinine, lactate dehydrogenase (LDH), ASAT and ALAT were determined by our hospital research facility.

### (Immuno)Histochemistry and antibodies

For (immuno)histological examination, 4μm paraffin sections were cut. Tubular injury was determined on sections stained with periodic acid Schiff after diastase (PasD). The percentage of damaged tubules in the corticomedullary region and in the outer cortex was estimated by a pathologist blinded for the groups using a 5-point scale according to the presence of necrosis in 10 randomly chosen non-overlapping high power fields (hpf, magnification 400x). Injury was graded as follows: 0 = 0%, 1 = <10%, 2 = 10–25%, 3 = 25–50%, 4 = 50–75%, and 5 = >75%. Immunohistochemical stainings to detect macrophages, neutrophils, apoptosis and proliferation were performed using rat-anti-mouse F4/80 (Serotec, Oxford, UK), FITC-labeled anti-mouse Ly-6G (BD Biosciences), rabbit-anti-mouse active caspase 3 (Cell Signaling Technology, Beverly, MA, USA) and rabbit-anti-human Ki67 (clone Sp6; Lab Vision Corp.) respectively. Macrophages and apoptotic and proliferating TEC were counted in the cortico-medullar region in 10 randomly chosen, non-overlapping hpf (magnification 400x). Total number of neutrophils was counted in one kidney sections per mouse.

### RNA Isolation and quantitative real-time RT-PCR

Total RNA was isolated from snap frozen kidney using the TRIzol^®^ reagent (Invitrogen, Breda, the Netherlands) method. Complementary DNA (cDNA) was synthesized using the M-MLV RT enzyme kit (Invitrogen), oligo dT primers (Sigma) and RNAse inhibitor (Applied Biosystem, Nieuwerkerk a/d IJssel, The Netherlands) according to the manufacturer’s protocol. Primer sequences were designed based on Primer3 software[23] and obtained from the Universal ProbeLibrary Assay Design Center (Roche): MCP-1 forward: catccacgtgttggctca; MCP-1 reverse: gatcatcttgctggtgaatgagt; KIM-1 forward: tggttgccttccgtgtctct; KIM-1 reverse: tcagctcgggaatgcacaa; NGAL forward: gcctcaaggacgacaacatc; NGAL reverse: ctgaaccattgggtctctgc; MCP-2 forward: tcagcccagagaagctgact; MCP-2 reverse: gggggatcttcagctttagtaca; MCP-3 forward: aggatctctgccacgcttc; MCP-3 reverse: ttgacatagcagcatgtggat; MCP-5 forward: ccaccatcagtcctcaggtatt; MCP-5 reverse: cggacgtgaatcttctgctt; iNOS forward: ctttgccacggacgagac; iNOS reverse: tcattgtactctgagggctgac; CCR7 forward: aaacccaggaaaaacgtgct; CCR7 reverse: acatgagaggcaggaaccag; ARG1 forward: ctccaagccaaagtccttagag; ARG1 reverse: aggagctgtcattagggacatc; YM1 forward: agaagggagtttcaaacctggt; YM1 reverse: gtcttgctcatgtgtgtaagtga; TBP forward: ggagaatcatggaccagaaca; and TBP reverse: gatgggaattccaggagtca. All primers were synthesized by Sigma (Zwijndrecht, the Netherlands).

Quantitative real-time RT-PCR was performed on a LightCycler® 480 System (Roche) using LightCycler® 480 SYBRGreen I Master mix (Roche). Forty-five cycles were carried out at an annealing temperature of 56–58°C. The starting concentrations of RNAs were calculated by the LinRegPCR program (version 9.30 beta) as described previously[24]. The expression of genes was normalized towards the reference gene TBP (TATA box binding protein).

### ELISAs

Snap frozen kidneys were homogenized in PBS containing 1% Triton X-100, 1mM EDTA, 1% protease inhibitor cocktail II (Sigma Chemicals). MPO, IL-1β, IL-6, IL-10, IL-17, Cxcl1, MCP-1, Cxcl2, and total and active TGFβ1 were measured in kidney homogenate using specific ELISAs (MPO: HyCult Biotechnology, Uden The Netherlands; others: R&D Systems) according to manufacturer instructions. MPO, cytokine and chemokine levels were corrected for the amount of total protein present using the Bio-Rad protein assay (Bio-RAD Laboratories) with IgG as standard. Plasma TNFα and IL-6 were analyzed using cytometric bead array (BD Biosciences) according to manufacturer’s instructions.

### Simulated ischemia/reperfusion in vitro

Primary mouse renal TEC from MCP-1^+/+^ and MCP-1^-/-^ were generated following the method described by Wuthrich *et al*.[25]. Briefly, the renal capsule was removed and tissue from the outer cortex was cut into pieces of approximately 1 mm^3^. Single cortical tubular cell suspensions of freshly dissected kidney cortices were prepared by collagenase type 1A (Sigma) dispersion at 37°C for 1 hour and washed in medium. TEC were subsequently grown to confluence on 6-well plates in HK2 medium (DMEM/F12 medium (Invitrogen) with 10% FCS (Hyclone), 5 μg/ml insulin and transferrin, 5 ng/ml sodium selenite (Roche), 20 ng/ml tri-iodo-thyrionine (Sigma), 50 ng/ml hydrocortisone (Sigma) and 5 ng/ml prostagladine E1 (Sigma) with L-glutamine and antibiotics (both from Invitrogen))

To simulate I/R *in vitro* TEC were placed in glucose-free HK2 medium and put in a hypoxic chamber. Chambers were purged for 10 minutes with a certified gas mixture consisting of 95% N_2_ and 5% CO_2_. Chambers were placed at 37°C for 1 hour, removed and re-purged for an additional 10 minutes and then returned to 37°C for 40 hours. Subsequently the cells were removed from the hypoxic chamber, supplemented with glucose and after 24 hours of reperfusion the amount of apoptotic and proliferating TEC was determined by flow cytometric analysis.

Apoptosis in TEC cultures was determined as described by Nicoletti *et al*.[26]. Briefly, TEC were incubated o/n at 4°C in 0.1% sodium citrate pH7.4 and 0.1% Triton X-100 supplemented with 50 μg/ml propidium iodide (PI; Molecular Probes Invitrogen, Breda, the Netherlands). To determine the percentage of proliferating TEC in cell culture, cells were fixed with 2% paraformaldehyde/PBS for 10 min on ice, permeabilized with 0.1% Triton X-100/PBS for 15 min at RT, incubated with Ki67 antibody (clone Sp6; Lab Vision Corp.) in 0.2% Tween20/PBS for 30 min on ice, and finally incubated with FITC-conjugated anti-rabbit for 30 min on ice. Apoptotic and proliferating TEC were analyzed by flow cytometry (FACSCalibur, Becton Dickinson, Franklin Lakes, NJ, USA).

### Statistical analysis

Results are expressed as mean ± standard error of the mean (SEM). Data were analyzed by Mann-Whitney U test, except data from the in vitro assays which were analyzed by unpaired Student’s *t* test. Kaplan Meier curve was used for the analysis of survival. Values of *P*≤0.05 were considered statistically significant. All statistical analyses were performed using GraphPad Prism4 (GraphPad Software, San Diego, California, USA).

## Results

### Renal MCP-1 is upregulated upon I/R injury

**Fig 1:**
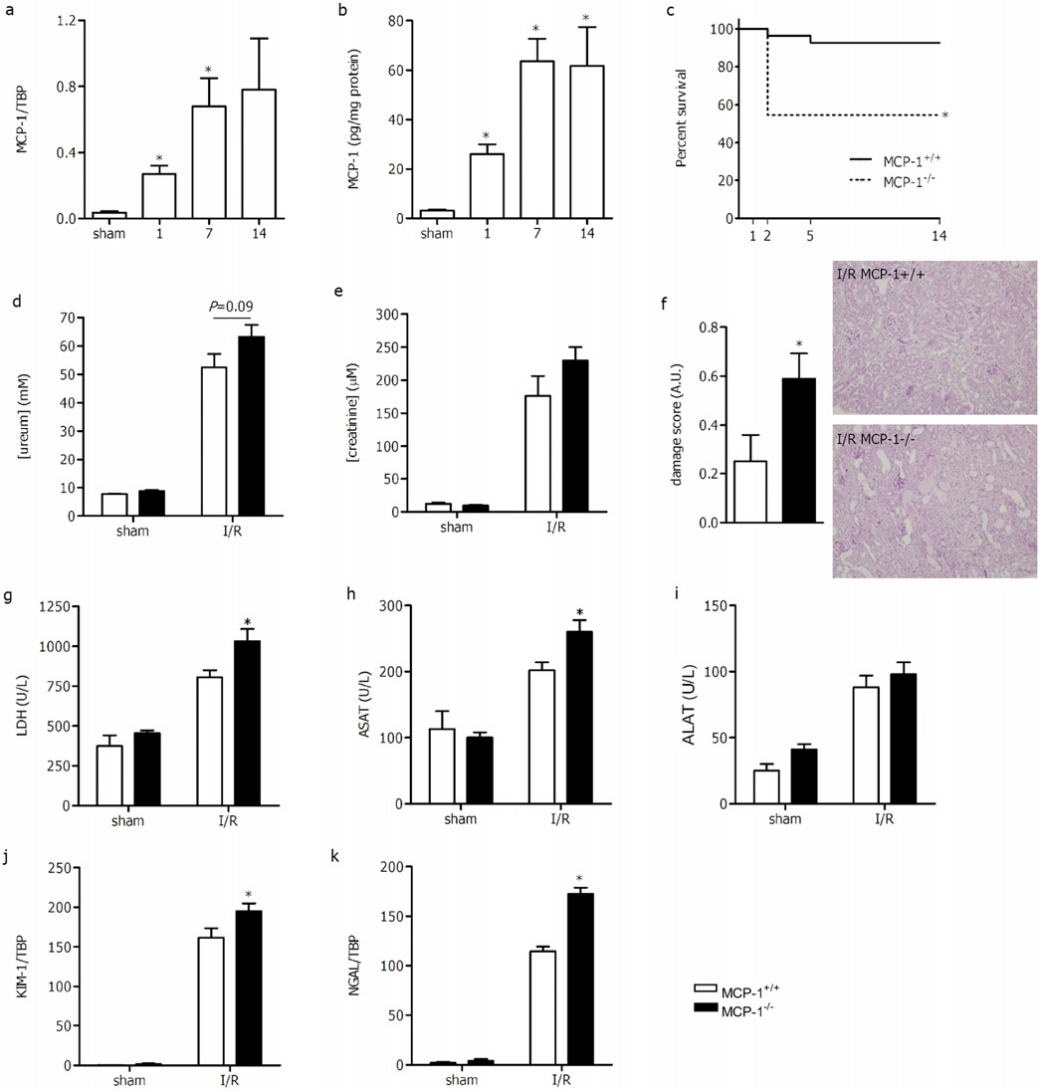
Renal MCP-1 expression 1, 7 and 14 days following I/R injury in MCP-1^+/+^ mice (white bars) on mRNA (a) and protein (b) level. (c) Kaplan-Meier survival curve revealed significant (*P* = 0.002) decreased survival in MCP-1^-/-^ compared with MCP-1^+/+^ mice following renal I/R injury. One day after renal ischemia/reperfusion injury (I/R) plasma ureum (d), and creatinine (e) were determined in MCP-1^+/+^ (white bars) and MCP-1^-/-^ (black bars). (f) Tubular damage was assessed in the outer cortex of MCP-1^-/-^ (white bars) and MCP-1^+/+^ (black bars) following renal I/R injury. Representative pictures of MCP-1^+/+^ and MCP-1^-/-^ PasD stained ischemic kidneys (10x magnification) are shown. Plasma levels of (g) LDH, (h) ASAT, and (i) ALAT were determined in sham and ischemic operated MCP-1^+/+^ (white bars) and MCP-1^-/-^ (black bars) mice. Renal expression of tubular injury markers kidney injury molecule-1 (KIM-1, j) and neutrophil gelatinase-associated lipocalin (NGAL, k) one day following ischemic injury (I/R) in MCP-1^+/+^ (white bars) and MCP-1^-/-^ (black bars) mice. Data are presented as mean ± SEM, n = 4–5 (sham), n = 9 (I/R), n = 22 (survival). *P<0.05 (a,b,d-k: Mann-Whitney U test; c: Kaplan Meier) compared with sham (a,b) or MCP-1^+/+^ (c-h).

MCP-1 mRNA expression upon renal I/R injury was significantly increased in kidneys of MCP-1^+/+^ mice at day 1 (7.6x ± 1.3) and reached a plateau at later time points (19.1x ± 4.7 at day 7 and 21.7 ± 8.6 at day 14, Fig 1a). The same pattern was observed for MCP-1 protein expression (Fig 1b). Already at day 1 MCP-1 protein in total kidney homogenate was significantly increased. Protein levels of MCP-1 continued to rise until day 7 and stayed elevated at day 14. Both mRNA and protein levels of MCP-1 are increased at day 1 and had reached a plateau at day 7 after renal I/R injury.

### MCP-1 deficiency increased lethality and renal damage

To study the role of MCP-1 in renal I/R injury, we subjected MCP-1^+/+^ and MCP-1^-/-^ mice to bilateral I/R injury. Surprisingly, MCP-1^-/-^ mice had a significantly decreased survival rate compared with MCP-1^+/+^ mice (Fig 1c). The induced renal injury is transient and does not result in significant lethality in MCP-1^+/+^ mice. However, 45% (10/22) of MCP-1^-/-^ mice died within 2 days after renal I/R injury.

Since MCP-1 deficiency affected I/R injury outcome significantly within the first 2 days, we next focused on the events that take place at day 1. Renal function was determined by measuring ureum and creatinine plasma levels (Fig 1d and 1e). There was a tendency (*P* = 0.09) towards higher ureum plasma concentration in MCP-1^-/-^ mice (63.2 ± 4.1mM) compared with MCP-1^+/+^ mice (52.4 ± 4.8mM). Creatinine plasma concentration was slightly but not significantly increased in MCP-1^-/-^ mice (230.0 ± 20.1μM) compared with MCP-1^+/+^ mice (176.2 ± 30.1μM). Ureum and creatinine levels were comparable between sham-operated mice of both strains.

Renal damage was assessed by scoring PasD-stained sections. Both mouse strains had severe tubular damage which was slightly higher in MCP-1^-/-^ compared with MCP-1^+/+^ mice (4.2 ± 0.10 versus 4.4 ± 0.05, *P*<0.05). Since tubular damage was already severe (maximum score is 5) in MCP-1^+/+^ mice, it is difficult to demonstrate an increase in damage in the corticomedullary region. As tubular damage can spread to the cortex in a severe model, we analyzed the outer cortex of the kidney in a similar fashion. Indeed, we observed mild (<10%) injury in the cortex of MCP-1^+/+^ mice which was significantly increased in the cortex of MCP-1^-/-^ mice (Fig 1f). Additionally, we analyzed the renal expression of kidney injury molecule-1(KIM-1) and neutrophil gelatinase-associated lipocalin (NGAL), two biomarkers for acute kidney injury[27;28]. Both KIM-1 and NGAL are significantly upregulated in the ischemic damaged kidney compared with sham kidney (Fig 1j and 1k). Moreover, following I/R injury MCP-1^-/-^ mice had significant increased levels of KIM-1 and NGAL compared with MCP-1^+/+^ mice. In line, LDH, a general damage marker reflecting necrosis, was significantly higher upon renal I/R injury in plasma of MCP-1^-/-^ mice compared with MCP-1^+/+^ (Fig 1f). In addition the liver damage markers ASAT was significantly higher in MCP-1^-/-^ mice compared with MCP-1^+/+^ mice following I/R injury (Fig 1h). Another marker of liver damage, ALAT had similar plasma levels in both strains (Fig 1i).

Overall, there is an increased expression of local and systemic damage markers in MCP-1^-/-^ mice which might explain the high mortality in these mice upon renal I/R injury.

### Altered macrophage polarization and increased renal MPO in MCP-1^-/-^ mice

**Fig 2:**
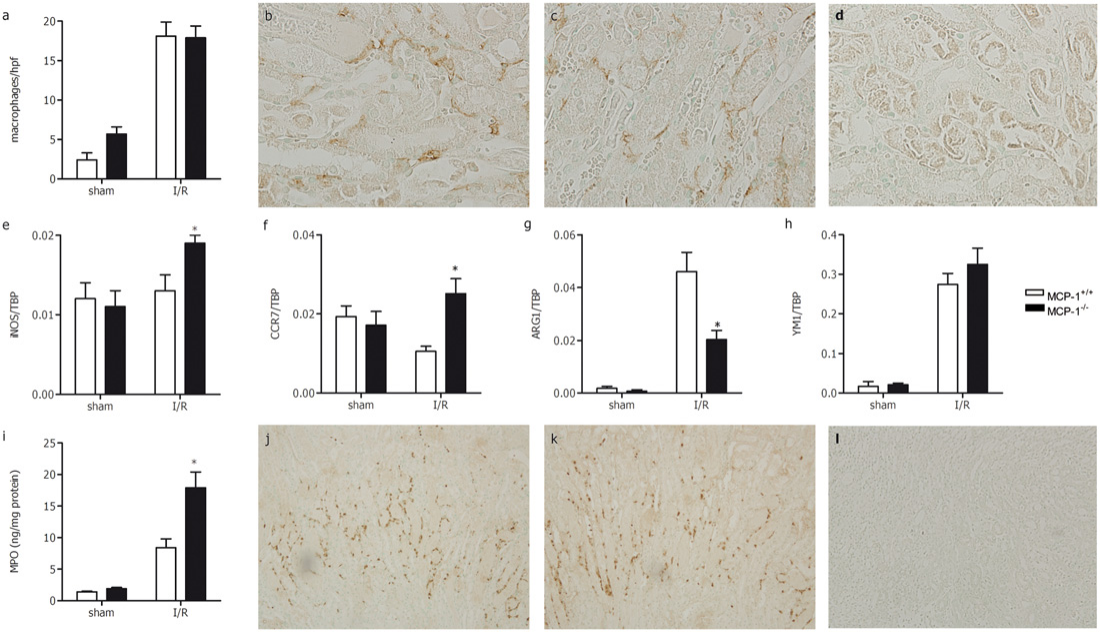
Leukocyte influx in kidneys from MCP-1^+/+^ (white bars) and MCP-1^-/-^ (black bars) mice 1 day after renal I/R or sham surgery. (a) The number of macrophages was determined in F4/80 stained paraffin kidney sections. Pictures (magnificantion 400x) of F4/80 stained kidney sections from MCP-1^+/+^ (b) and MCP-1^-/-^ (c) after I/R injury. (d) Negative control of F4/80 staining. Renal mRNA expression of M1 macrophage markers iNOS (e) and CCR7 (f), and the M2 macrophage markers ARG1 (g) and YM1 (h) were determined in sham and I/R MCP-1^+/+^ and MCP-1^-/-^ mice. (i) Renal MPO was significant increased in MCP-1^-/-^ compared with MCP-1^+/+^ mice after I/R injury. However, Ly6-G stained kidneys sections (magnification 100x) revealed no difference between the total number of neutrophils in MCP-1^+/+^ (j) and MCP-1^-/-^ (k) mice after I/R. (l) Negative control of Ly6-G staining. Data are presented as mean ± SEM, n = 4–5 (sham) and n = 9 (I/R). *P<0.05 (Mann-Whitney U test).

MCP-1 is the main chemoattractant for monocytes; despite functional redundancy with other chemokines *in vitro*, MCP-1 alone is responsible for mononuclear cell infiltration in several inflammatory models *in vivo*[6–9]. One day after renal I/R injury macrophage accumulation, determined by scoring F4/80 stained paraffin kidney sections, was significantly increased in both MCP-1^+/+^ and MCP-1^-/-^ compared with sham-operated mice (Fig 2a–2c). No difference in macrophage accumulation between both strains was observed. To determine whether there was a difference in macrophage polarization, we analyzed the expression of iNOS and CCR7, two markers of M1 macrophages[29], and the expression of ARG1 and YM1, two markers of M2 macrophages[29], in the kidney. In sham-operated kidneys these macrophage polarization markers were comparable between MCP-1^+/+^ and MCP-1^-/-^ mice. Both M1 markers iNOS and CCR7 were significantly enhanced, while the M2 marker ARG1 was significantly reduced in MCP-1^-/-^ mice upon renal I/R injury compared with MCP-1^+/+^ (Fig 2e–2g). Expression of YM1 was similar between both strains (Fig 2h). This indicates that, although equal numbers of macrophages were present, the balance has shifted towards a more pro-inflammatory (M1) macrophage in MCP-1^-/-^ mice.

Influx and activity of neutrophils was determined by immunohistochemistry and myeloperoxidase (MPO) ELISA respectively. Immunohistochemical staining revealed equal numbers of neutrophils in MCP-1^+/+^ (502 ± 52) and MCP-1^-/-^ (479 ± 50) after I/R (Fig 2j and 2k). Both mouse strains had increased levels of renal MPO in I/R kidneys as compared with sham-operated kidneys. Interestingly, significantly more MPO was present in MCP-1^-/-^ mice 1 day after renal I/R injury compared with MCP-1^+/+^ mice (Fig 2i).

### Renal cytokine and chemokine expression

The balance between pro- and anti-inflammatory mediators might be dysregulated in MCP-1^-/-^ mice subjected to renal I/R. However, no difference in renal IL-6, IL17, KC, MIP-2, and IL-10 levels, or in plasma TNFα and IL-6 between both strains was observed post-ischemia (table 1). Slightly, but significant, lower IL-1β levels in MCP-1^-/-^ mice were detected in renal homogenates after I/R compared with MCP-1^+/+^ mice (4.5 ± 0.5 (MCP-1^+/+^) and 3.1 ± 0.3 (MCP-1^-/-^) pg IL-1β/mg protein, *P*<0.05; table 1). To investigate whether compensatory expression of other monocyte chemoattractant chemokines was present in MCP-1^-/-^ mice, MCP-2, MCP-3 and MCP-5 mRNA levels were determined by quantitative real-time RT-PCR. Of note, deficiency for MCP-1 was confirmed in sham and ischemic MCP-1^-/-^ kidneys by quantitative real-time RT-PCR. No difference in renal MCP-2 (below detection level) and MCP-5 (MCP-1^+/+^ 0.85 ± 0.29; MCP-1^-/-^ 0.46 ± 0.27 AU) expression was found between both strains 1 day after renal I/R injury. Following I/R injury, renal MCP-3 mRNA expression was significantly lower in MCP-1^-/-^ mice (MCP-1^+/+^ 0.557 ± 0.094; MCP-1^-/-^ 0.153 ± 0.021, *P*<0.05).

**Table 1:** Cytokine levels post-ischemia.

		MCP-1^+/+^	MCP-1^-/-^
Kidney (pg/mg total protein)	KC	133.9 ± 14.7	114.5 ± 10.9
	Cxcl2	24.8 ± 3.6	22.8 ± 1.7
	IL-1β	4.5 ± 0.5	3.1 ± 0.3*
	IL-6	23.9 ± 1.9	22.3 ± 2.0
	IL-10	5.87 ± 0.46	5.75 ± 0.62
	IL-17	7.53 ± 1.19	9.34 ± 2.27
Plasma (pg/ml)	TNFα	1.7 ± 0.1	1.6 ± 0.1
	IL-6	6.1 ± 1.1	5.8 ± 0.9

Data is expressed as mean ± sem.

*P<0.05.

### MCP-1 deficiency induces tubular epithelial apoptosis but tubular epithelial proliferation is unaffected

**Fig 3:**
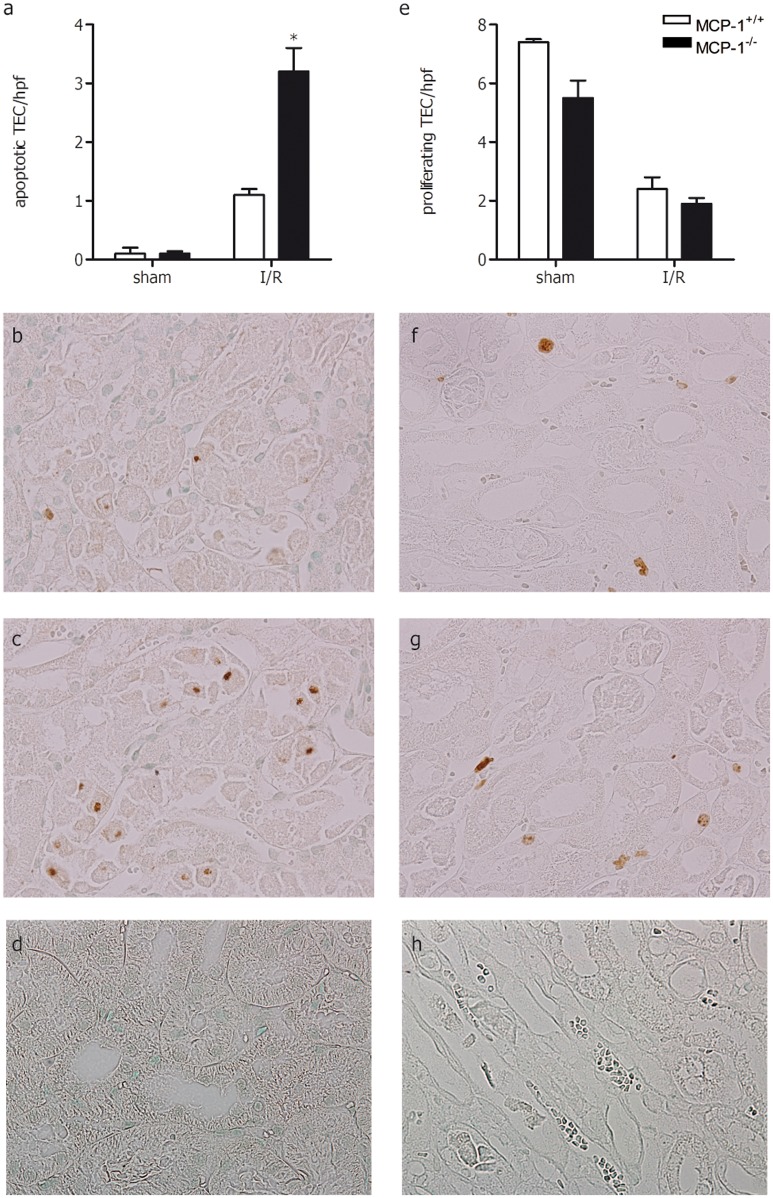
Apoptotic and proliferating TEC in kidneys from MCP-1^+/+^ (white bars) and MCP-1^-/-^ (black bars) mice 1 day after renal I/R injury or sham surgery. (a) The number of apoptotic TEC was quantified in caspase-3 stained paraffin kidney sections. Pictures (magnification 400x) of caspase-3 stained kidney sections from MCP-1^+/+^ (b) and MCP-1^-/-^ (c) mice after renal I/R injury. (d) Negative control of caspase-3 staining. (e) The number of proliferating TEC was determined in Ki67 stained paraffin kidney sections. Pictures (magnification 400x) of Ki67 stained kidney sections from MCP-1^+/+^ (f) and MCP-1^-/-^ (g) mice after renal I/R injury. (h) Negative control of Ki67 staining. Data are presented as mean ± SEM, n = 4–5 (sham) and n = 9 (I/R). *P<0.05 (Mann-Whitney U test).

To investigate whether MCP-1 deficiency has an effect on TEC apoptosis, the amount of tubular apoptosis during renal I/R injury was assessed by scoring immunohistochemical stainings for caspase-3. Compared with sham-operated mice, animals subjected to renal I/R injury demonstrated significant increased TEC apoptosis (Fig 3a). Interestingly, MCP-1^-/-^ mice had a 3-fold increase in TEC apoptosis compared with MCP-1^+/+^ mice after renal I/R injury (Fig 3a–3c).

**Fig 4:**
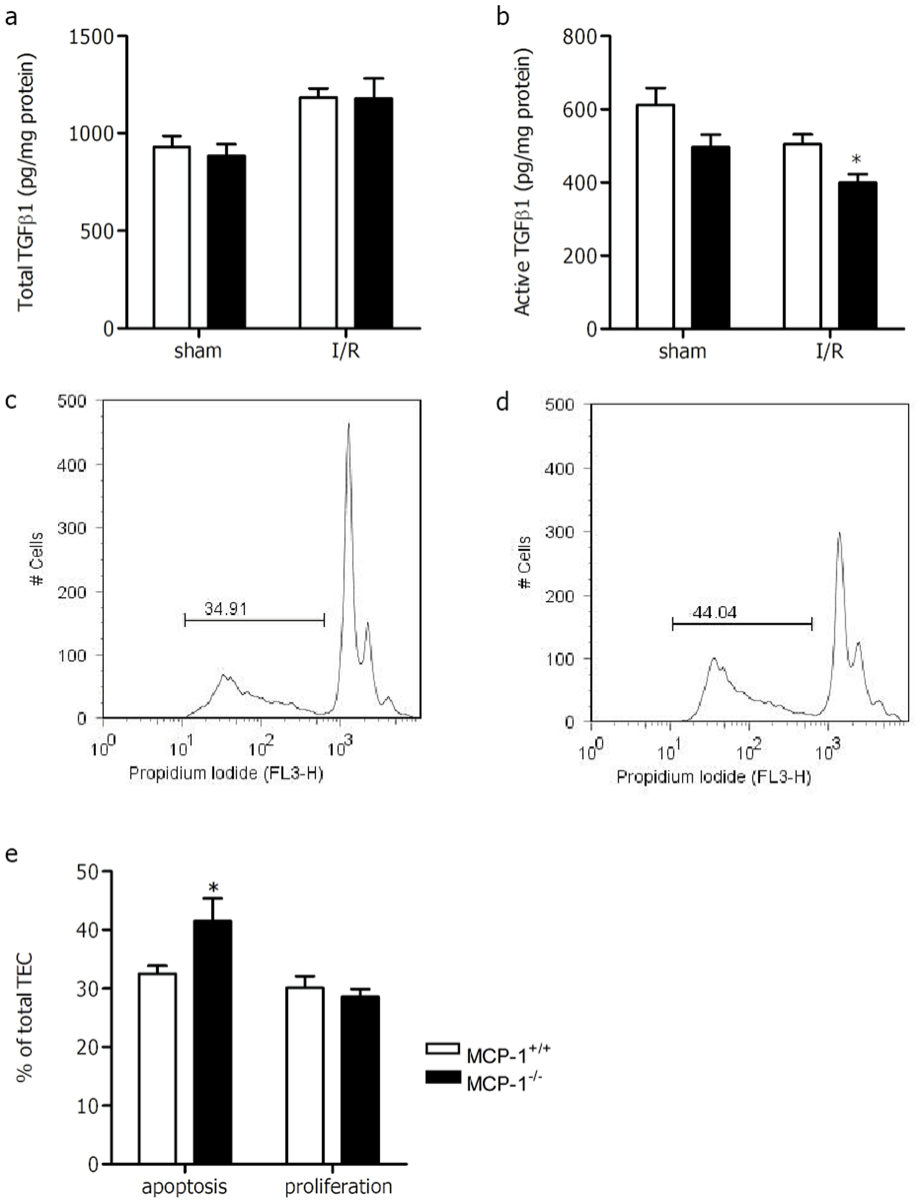
Renal TGFβ1 and apoptosis and proliferation of TEC after simulated I/R in vitro. Total (a) and active (b) TGFβ1 was determined in kidneys of sham and ischemic operated MCP-1^+/+^ (white bars) and MCP-1^-/-^ (black bars). (c-e)TEC were isolated from MCP-1^+/+^ and MCP-1^-/-^ kidneys and grown to confluence. Subsequently TEC were subjected to simulated ischemia/reperfusion. Flowcytometry histograms of MCP-1^+/+^ (c) and MCP-1^-/-^ (d) TEC; isolated nuclei were analyzed for propidium iodide staining of DNA. The percentage of apoptotic nuclei (broad hypodiploid peak) is given as a percentage of total TEC. The narrow peak (diploid) represents viable cells. (e) Graphic representation of the percentage of apoptotic and proliferating TEC of MCP-1^+/+^ (white bars) and MCP-1^-/-^ (black bars) mice. Data are presented as mean ± SEM, n = 4–5 (sham) and n = 9 (I/R). or pooled from two independent experiments (in vitro) (n = 5–6). *P<0.05 (Mann-Whitney U test (a-b) or unpaired Student’s *t* test).

In order to better understand the MCP-1-mediated difference in cell death/survival, we next analysed TGFβ1 as this protein is involved in the survival of epithelial cells[30;31] and its absence enhances renal I/R injury in mice[32]. For this we determined both total and active TGFβ1 in kidney homogenate. No difference in total TGFβ1 (Fig 4a) was observed between both strains, however, active TGFβ1 (Fig 4b) was significantly lower in MCP-1^-/-^ kidneys following I/R injury.

Next we investigated the proliferation of TEC by determining the numbers of Ki67 positive TEC in the kidney. No differences were observed in the number of proliferating TEC between sham-operated and post-ischemic kidneys from MCP-1^+/+^ and MCP-1^-/-^ (Fig 3e–3g).

### Tubular epithelial apoptosis is enhanced in vitro in MCP-1 deficient TEC after simulated I/R

To determine whether MCP-1 directly affects tubular epithelial apoptosis, TEC were isolated from MCP-1^+/+^ and MCP-1^-/-^ kidneys and subjected to simulated I/R *in vitro*. In line with the *in vivo* experiments, apoptosis was significantly enhanced in MCP-1^-/-^ TEC compared with MCP-1^+/+^ TEC (Fig 4c–4e). The percentage of proliferating TEC was similar between both groups (Fig 4c). These results indicate that MCP-1 directly affects TEC by enhancing its survival.

## Discussion

Chemokines are important players in the complex pathogenesis of renal I/R injury. In the present study we focused on the role of the MCP-1 during ischemic renal injury. Besides serving as a chemoattractant for inflammatory cells, primarily monocytes, MCP-1 has been shown to possess additional functions. Indeed, this study shows, to our knowledge, for the first time that MCP-1 is essential for TEC survival. Additionally, we show a role for MCP-1 in the polarization of macrophages upon renal injury.

*In vitro*, renal TEC produce MCP-1 in response to various stimuli[1] including ischemia/reperfusion injury[33]. Accordingly, we and others[2;3;34;35] see an increase of MCP-1 both on mRNA and protein level after inducing renal I/R injury coinciding with the accumulation of macrophages[3;34]. Variations in kinetics of MCP-1 expression might be explained by the different animal models used. Typically, macrophage accumulation in renal I/R injury is observed in the reparative phase following the initial inflammatory response characterized by massive influx of neutrophils[34]. Surprisingly, MCP-1 deficiency leads to lethality during the inflammatory response early after inducing I/R injury.

To clarify this unexpected high mortality early after renal I/R injury in MCP-1^-/-^ mice, we focused on the inflammatory mechanisms taking place prior to mortality. Although renal dysfunction was not significantly different between both mouse strains, local (Kim-1 and NGAL) and systemic (plasma LDH) damage markers were significantly increased in MCP-1^-/-^ mice compared with MCP-1^+/+^ mice. Renal injury markers Kim-1[36] and NGAL[37] are potential biomarkers that predict tubular damage at an earlier stage than the routinely used plasma markers ureum and creatinine. In addition to elevated damage markers, tubular apoptosis in the post-ischemic kidney was markedly enhanced in MCP-1^-/-^ compared with MCP-1^+/+^. Altogether this explains to a large extent the high mortality in MCP-1^-/-^ mice.

One day after renal I/R injury leukocyte influx consists predominantly of neutrophils[34]. Neutralizing Cxcl1 or Cxcl2, the main neutrophil chemoattractants in I/R injury, attenuates renal I/R injury with concomitant reduced influx of neutrophils[38]. Both renal Cxcl1 and Cxcl2 protein levels are elevated in MCP-1^+/+^ and MCP-1^-/-^ mice upon I/R injury, suggesting that these two chemokines are responsible for neutrophil influx. In addition to MCP-1 other monocyte chemoattractant proteins (such as MCP-2/Ccl8 and MCP-5/Ccl12)[21] are able to induce the accumulation of macrophages in injured tissue. We have previously shown that MCP-5 expression is increased in the ischemic kidney[34].

The inflammatory response upon renal I/R injury in MCP-1^-/-^ mice was accompanied by increased MPO while the number of neutrophils was not altered. The enzyme MPO is mostly related to neutrophils and to a lesser extent to monocytes, however during monocyte maturation into macrophages MPO is usually lost[39]. Increased MPO levels were also observed in MCP-1^-/-^ mice subjected to toxic liver injury[40] and skeletal muscle ischemia[8], however the number of neutrophils was not reported in these studies. Our data could indicate that MCP-1^-/-^ neutrophils produce more MPO and as a consequence can initiate, once released extracellularly, tissue damage via the generation of the conversion of hydrogen peroxide (H_2_O_2_) into the hyperreactive radical hypochlorous acid (HOCl).

A direct effect of MCP-1 on survival has been shown in various cell lines and primary cells[13–16;18]. Indeed, MCP-1 deficient TEC are more vulnerable to *in vitro* simulated I/R cell death. Furthermore, the balance between apoptotic and proliferating TEC was shifted in MCP-1^-/-^ mice; more apoptotic TEC were present after I/R while the number of proliferating TEC was similar as in MCP-1^+/+^ mice. In accordance, Narasaraju *et al*. showed that MCP-1 antibody treatment increases apoptosis of alveolar epithelial cells in influenza pneumonitis[17]. One could argue that a defective clearance of damaged cells due to an impaired chemotaxis of monocytes is responsible for the increased apoptosis. However, in the present study macrophage accumulation is not altered and hence increased TEC apoptosis is not likely due to a defect in monocyte chemotaxis. Another possible mechanism could be TGFβ1 pathway which is reduced in our study in MCP-1^-/-^ mice and has been reported to play a role as a survival factor for TEC[32].

Interestingly, upon renal I/R injury there was a shift in macrophage type towards a more pro-inflammatory (M1) phenotype in MCP-1^-/-^ mice compared with MCP-1^+/+^. A role for MCP-1 in macrophage polarization has been suggested by others[18;41;42]. Blocking MCP-1 reduced M2 macrophage number in tumors *in* vivo[41]. In addition, neutralization of Ccl2 resulted in impaired expression of M2 polarization-associated markers and enhanced expression of M1 polarization-specific genes in human macrophages[42]. Moreover, Roca *et al*. demonstrate that MCP-1 induces the activation of M2 macrophages[18]. This suggests that MCP-1 deficiency skews the macrophage polarization towards M1 due to a reduced ability to differentiate into M2 macrophages.

Although blocking the Ccr2 receptor prevented renal damage with concomitant reduced numbers of infiltrating leukocytes during renal I/R injury[19;20], we found that MCP-1 deficiency severely induces renal damage without affecting the number of infiltrating leukocytes. This dissimilarity could be explained by the fact that blockage of Ccr2 prevents the interaction of different chemokines with their receptor, while in our study we determined the role of MCP-1 which might have functions independent of its interaction with Ccr2. MCP-1 deficiency did not result in compensatory expression of other known Ccr2 ligands. On the contrary, MCP-3 was significantly lower in MCP-1^-/-^ ischemic kidneys suggesting that Ccr2 signaling in MCP-1^-/-^ mice is additionally decreased by reduced MCP-3 expression. Although blocking the Ccr2 receptor prevented renal damage[19;20], reduced Ccr2 signaling in MCP-1 deficient mice did not. These results would argue for a Ccr2-independent role of MCP-1. Indeed, our data indicate that the effect of MCP-1 goes beyond its chemoattractant properties for leukocytes. In addition, Ccr2-independent MCP-1 interactions have been reported by Schecter *et al* who showed that Ccr2 deficient smooth muscle cells can still be activated by MCP-1[43]. We propose that MCP-1 reduces necrosis and apoptosis of TEC during renal I/R injury. In addition, MCP-1 might play a role in the polarization of macrophages towards the anti-inflammatory (M2) phenotype. The results of our study indicate that MCP-1 has versatile functions during different phases of renal I/R injury. It is conceivable that MCP-1 has a time and spatial dependent expression whereby blocking MCP-1 early after I/R may be detrimental due to increased tissue damage. Due to its versatile functions, caution has to be taken when considering MCP-1 as a therapeutic target in renal disease.

## Supporting Information

S1 ARRIVE Checklist (PDF)
